# Semi-automated micro-computed tomography lung segmentation and analysis in mouse models

**DOI:** 10.1016/j.mex.2023.102198

**Published:** 2023-04-20

**Authors:** Jonathan D. Luisi, Jonathan L. Lin, Lorenzo F. Ochoa, Ryan J. McAuley, Madison G. Tanner, Obada Alfarawati, Casey W. Wright, Gracie Vargas, Massoud Motamedi, Bill T. Ameredes

**Affiliations:** University of Texas Medical Branch, 301 University Blvd., Galveston, TX 77555, United States

**Keywords:** Image analysis, Automated Image Segmentation, Manual image segmentation, Bleomycin, Pulmonary toxicology, Lung tissue density, Semiautomated Micro-CT Lung Segmentation and Analysis in Mouse Models

## Abstract

Computed Tomography (CT) is a standard clinical tool utilized to diagnose known lung pathologies based on established grading methods. However, for preclinical trials and toxicity investigations in animal models, more comprehensive datasets are typically needed to determine discriminative features between experimental treatments, which oftentimes require analysis of multiple images and their associated differential quantification using manual segmentation methods. Furthermore, for manual segmentation of image data, three or more readers is the gold standard of analysis, but this requirement can be time-consuming and inefficient, depending on variability due to reader bias. In previous papers, microCT image manual segmentation was a valuable tool for assessment of lung pathology in several animal models; however, the manual segmentation approach and the commercial software used was typically a major rate-limiting step. To improve the efficiency, the semi-manual segmentation method was streamlined, and a semi-automated segmentation process was developed to produce:

• Quantifiable segmentations: using manual and semi-automated analysis methods for assessing experimental injury and toxicity models,
• Deterministic results and efficiency through automation in an unbiased and parameter free process, thereby reducing reader variance, user time, and increases throughput in data analysis,
• Cost-Effectiveness: portable with low computational resource demand, based on a cross-platform open-source ImageJ program.

## Graphical abstract

**Figure:**
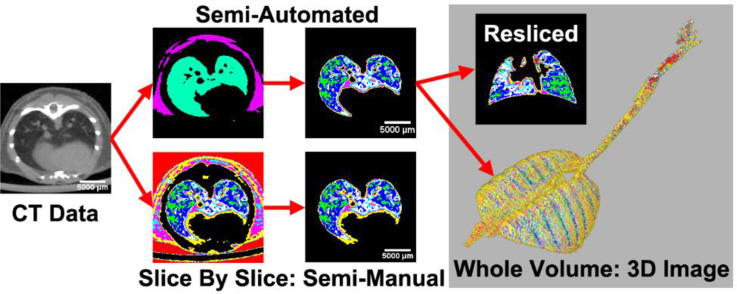

Specifications table

**Table:** 

Subject area:	Pharmacology, Toxicology and Pharmaceutical Science
More specific subject area:	*Pulmonary Toxicology*
Name of your method:	Semiautomated Micro-CT Lung Segmentation and Analysis in Mouse Models
Name and reference of original method:	Tian, B., Liu, Z., Litvinov, J., Maroto, R., Jamaluddin, M., Rytting, E., Patrikeev, I., Ochoa, L., Vargas, G., Motamedi, M., Ameredes, B.T., Zhou, J., Brasier, A.R., 2019. Efficacy of Novel Highly Specific Bromodomain-Containing Protein 4 Inhibitors in Innate Inflammation–Driven Airway Remodelling. Am. J. Respir. Cell Mol. Biol. 60, 68–83. 10.1165/rcmb.2017–0445OC
Resource availability:	*Computed Tomography Images* *NIH's ImageJ* *https://github.com/UTMB-Luisi/SemiAutomated_Lung_CT*

## Method details

Preclinical animal studies are critical to the understanding of inflammatory processes and resulting injury. With lung toxicant exposures via aerosols or aspiration, there is a progression of inflammatory responses that can lead to specific pathological presentations. Biopsy histology and bronchoalveolar lavage fluid (BALF) samples are among the analytical procedures used in preclinical research settings; however, chest CT imaging is used in the clinic as a less invasive method to detect pathology, and is translationally relevant to preclinical animal studies [1], [2], [3], [4], [5], [6]. For well-studied clinical pathologies, there are established clinical grading systems based on specific image features such as ground-glass opacities, as seen in Electronic Cigarette or vaping-use associated lung injury (EVALI), and the lungs of SARS-Co-V-2 patients [2,7,8]. Pulmonary emphysema, for example, has a standard grading system involving a workflow of fully automated threshold segmentation and analysis routines [4]. However, there are no standard objective grading processes for use in experimental animal models of lung disease, namely those in which agents such as saline, polyinosinic-polycytidylic acid (Poly-IC), or bleomycin are administered intratracheally or intranasally [9,10]. A system to discover and evaluate correlated tissue image features for specific pathologies is desirable for analysis methodology [11], [12], [13]. Usually, evaluation of image features in injury and disease models is achieved by manual segmentation and grading images. For example, in a prior study [14], Poly-IC, a viral mRNA pathogen associated molecular pattern, was investigated as an agent producing lung injury and fibrosis in mice, in which detectable alterations were observed in lung tissue/air radiodensity ratios in the −1000 to −200 Hounsfield units (HU, when calibrated with air and water references −500HU is 50/50 tissue to air ratio) range, using the manual segmentation method. Based on those data, we developed a semi-automated ImageJ macro for unbiased and reproducible segmentation and quantitative radiodensity analysis of lung pathology, with minimal supervision necessary in the validation steps, in order to reduce the effort of manual image segmentation and grading. The user-necessary input was reduced to validation of an automatically segmented connected-components image, which was utilized as a mask for automated radiodensity and volume calculation of subsequent images by the computer. The goal of this analysis pipeline was to deliver a low- cost, efficient, accessible, and reproducible solution to 3D lung image quantification through automation.

The balance of automation and user supervision is important in experimental studies, as a user can identify sources of variance and determine if it is measurement drift, experimental error, or indication of new features of pathology. While a fully automated system is desirable to limit the time and knowledge that a reader needs to efficiently process image data, manual checkpoints provide minimal user intervention to validate the output. Computational efficiency also can limit the time to process, and is impacted by the availability of computing resources, i.e., RAM size and CPU clock-speeds. While a GPU-optimized algorithm can exceed CPU computation, the scale of image data requires larger GPUs and often employs down-sampling methods. While fully automated Deep Learning algorithms such as Convolutional Neural Networks (CNN) are gaining popularity, many labs do not have the high GPU/RAM based supercomputers capable of handling image data [15], [16], [17], [18], [19]. Other methods, such as active contours offer interactive tuning for better manual segmentations; however, the method may require human refinement [20]. While we did assess some commercial systems with license or subscription requirements, we chose to develop an algorithm using the NIH-funded ImageJ to achieve a balance of speed, efficiency, and broad availability. As an ImageJ macro set, these methods are portable and computationally efficient to run on a wide range of commonly available computer hardware. Furthermore, the ImageJ program FIJI supports Windows, Mac OS, and Linux, maximizing potential utilization across operating systems and hardware platforms.

A Siemens Micro-CT imaging system (Inveon CT/PET/SPECT micro-computed tomography small animal scanner Siemens, Germany) was utilized to capture whole body volumes of mice. Calibration of the radiodensity, with air and water as reference materials, was completed before imaging, per the manufacturer's instructions. CT acquisition parameters of 1000 ms (1 s) per projection, 70 kV X-ray source voltage, 500uA X-ray source current, 107 μm isotropic resolution, 360° rotation with 520 projections were used. The resulting image was 54.85 × 54.85 × 82.27 mm field-of-view at scanning, containing 512 × 512 × 768 voxels of reconstructed image. In the original paper, the software used was Siemens Inveon Image Acquisition Workplace and Inveon Image Research Workplace, to manually segment the lung segments. From the related paper [14], HU bins of [−1000 to −600, −600 to −500, −500 to −400, −400 to −300, −300 to −200, −200 to 0] were used to segment the lung radiodensity when manually selected. Within this HU bin manual segmentation construct, the bleomycin lung injury model demonstrated a right-shifted HU density histogram when the lungs were experimentally driven toward the fibrotic stage; accordingly, through automation, we aimed to replicate that finding in full 3D, with minimal user interactions [21].

**Fig. 1:**
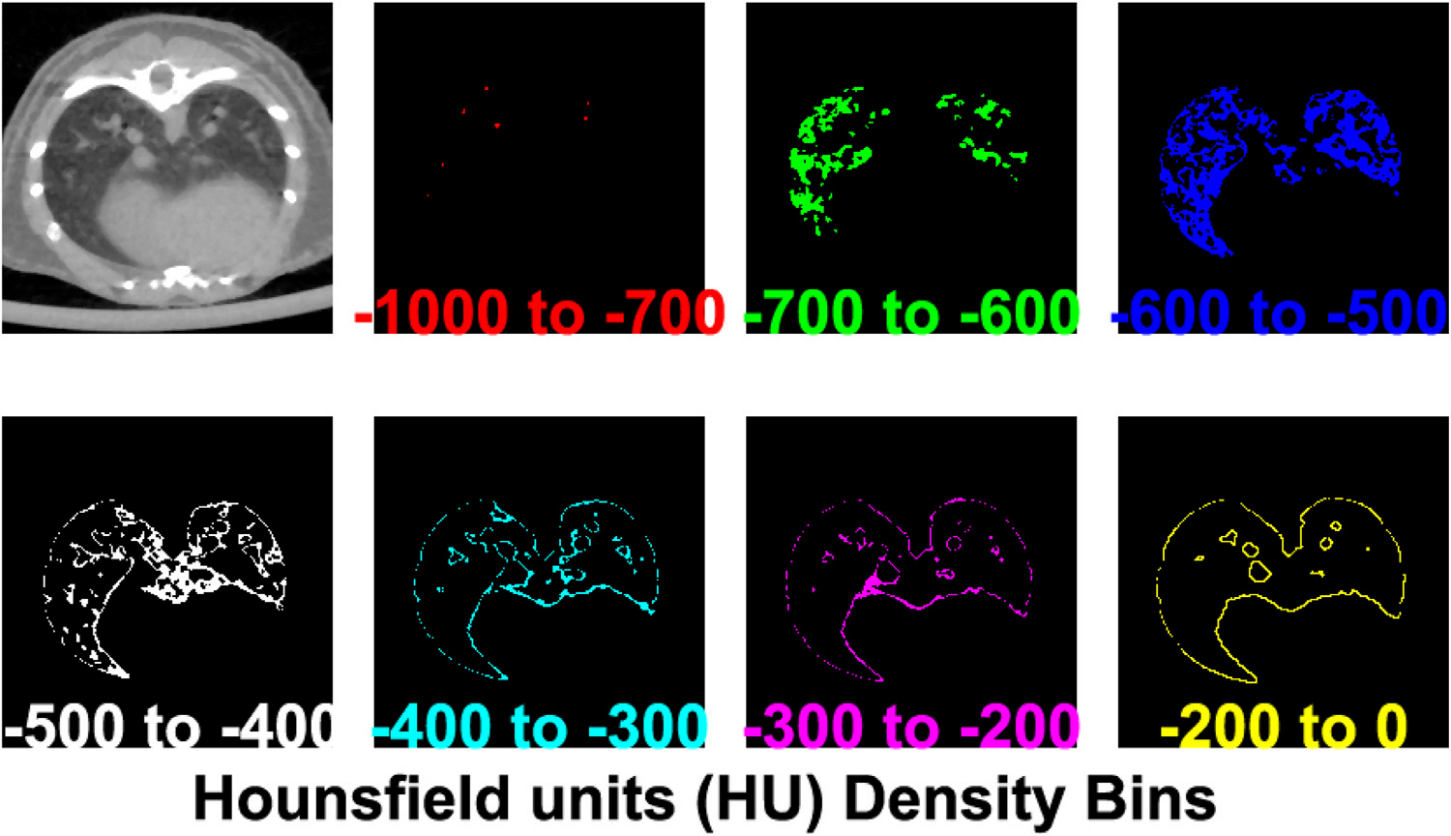
The Hounsfield Units (HU) that correspond to the lung tissue and connective tissue are color coded by intensity bin. Each bin range shows a unique portion of the lung morphology. Key bins are [−1000 to −700] corresponding to regions of air (e.g. lumen of large airways in this CT cross sectional slice), [−500] HU corresponding to areas having a 50/50 ratio of air/tissue, and [−200 to 0] for connective tissue.

The HU bin selection is important to properly segment lung tissue. Accordingly, modifications for the semi-manual and automated segmentation were based on the prior two papers [9,22]; however, the methods correlate with other findings and devices. In another study [23], the software Amira (FEI, Hillsboro, Oregon, USA) was used for semi-automated segmentation by the “magic wand” tool, a manual nearest-neighbor connected-components tool, using a HU range of [−1000 to −150] to roughly segment the lung, and then morphological operators were applied to refine the model. An alternative segmentation approach using deep learning [17] used a threshold of −200 HU lung for segmentation in a normal and bleomycin induced injury model. Rather than set a single broad range, in our analysis of experimentally-induced lung damage, lung tissue density specificity in the air-alveolar tissue-associated HU range from [−1000 to −500] was split into sub-ranges of [−1000 to −700, −700 to −600, and −600 to −500] HU, to allow for increased specificity, as shown in Fig. 1.

To avoid portability issues across operating systems and hardware platforms, ImageJ (version 1.52 h) was used; and while the scripts currently look for “.HDR” filetypes, ImageJ can also import generic DICOM data as well. The data was imported into ImageJ with the Bioformats importer as 16-bit float images. For our comparison between the semi-manual segmentation versus the automated method, there was no pre-processing of the data in this application.

The ImageJ macros can be found in the supplement and: https://github.com/UTMB-Luisi/SemiAutomated_Lung_CT.

### Semi-manual segmentation

Segmentation runs automatically with a macro “CT_AutoHU_SelectBin.ijm,” which allows for automated and batch processing of images. There are three modes: (1) for HU bins defined as “Default” (as used in the original papers [14,21], (2) “Mod” with the subranges split for more accuracy, and (3) “Range,” where a max, min, and bin width can be user defined.

**Fig. 2:**
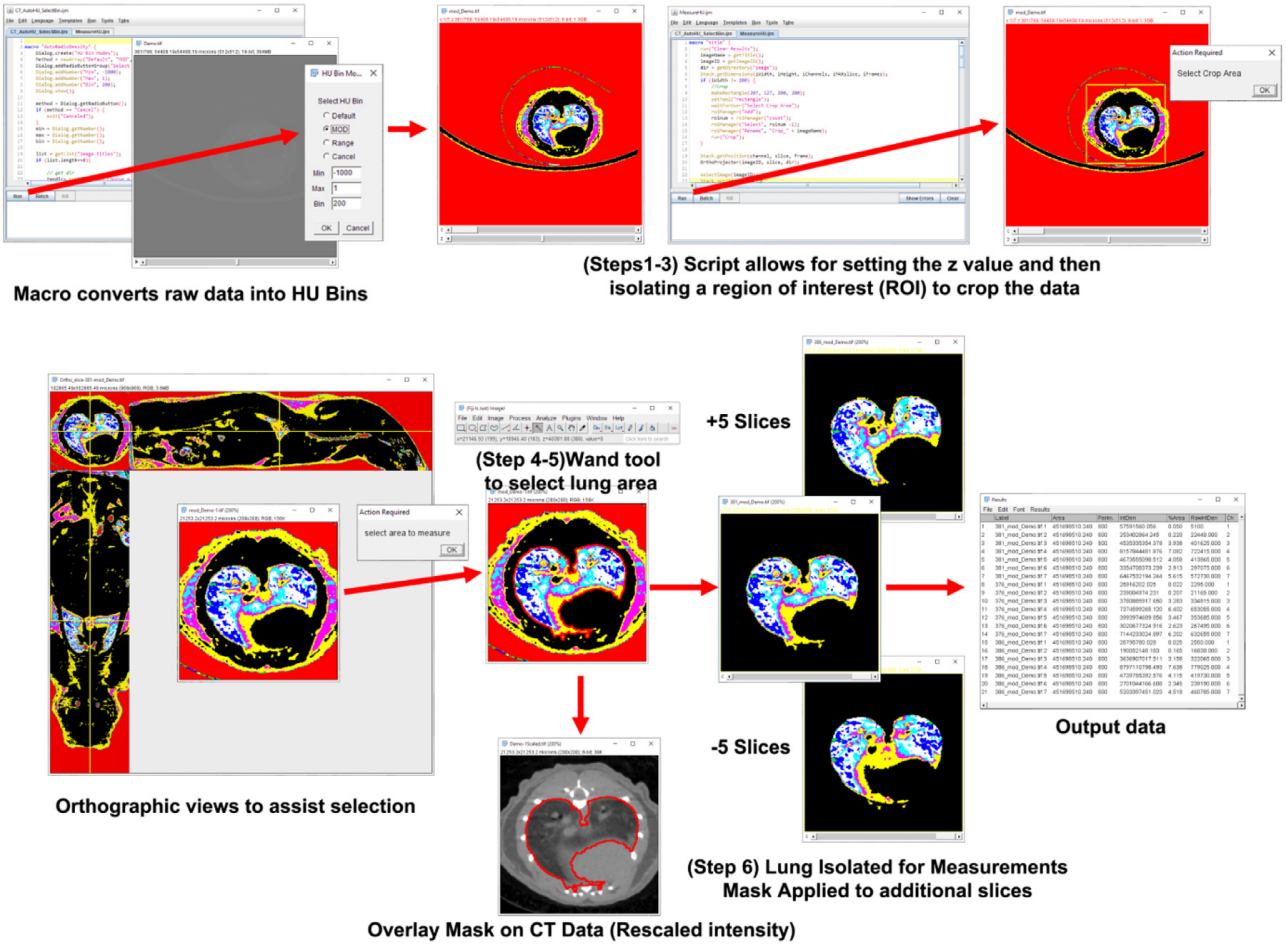
Workflow of the manual segmentation process that can guide the user through transforming the CT data into HU bins and selecting the lung from surrounding tissue. The first steps, 1–3, can be run in batch mode, while the semi-manual segmentation was steps 4–6. The Macro automatically quantifies the selected slice, and then quantifies 5 slices above and below the selected slice, to augment the measuring power. All critical steps are maintained, so that the analyst's work can be tracked, i.e., the selection area can be overlaid on the original data, in order to validate the segmentation.

After the initial HU bin segmentation, the .TIFF files are readied for manual segmentation using the script “MeasureHU.IJM”. The procedure illustrated in Fig. 2, is as follows:

(1) Drag the z-axis slider in the bottom of the ImageJ window and pick specified slice (shown top left Z: current/total slices
(2) Run the macro MeasureHU.IJM from editor by clicking “Run” button
(3) To select the crop area, drag the region of interest (ROI) selection to center the lung and click ok on the popup window
(4) Two new windows will open, an orthographic view to reference the lung and a color image of the lung segmentation.
(5) Select the color image using the wand tool, “Shift + left click” to select the lung
  (I) To Zoom: “CTRL + Wheel”
  (II) To Pan: “Space + drag”
  (III) To make selection easier to see “CTRL + Y”
    (a) Set stroke color to “red”
    (b) Set with to “0.5″
  (IV) In the dialog “select area to measure” then click ok when all the lung is selected
(6) Images and results table are automatically saved

### Semi-automated connected-components segmentation

**Fig. 3:**
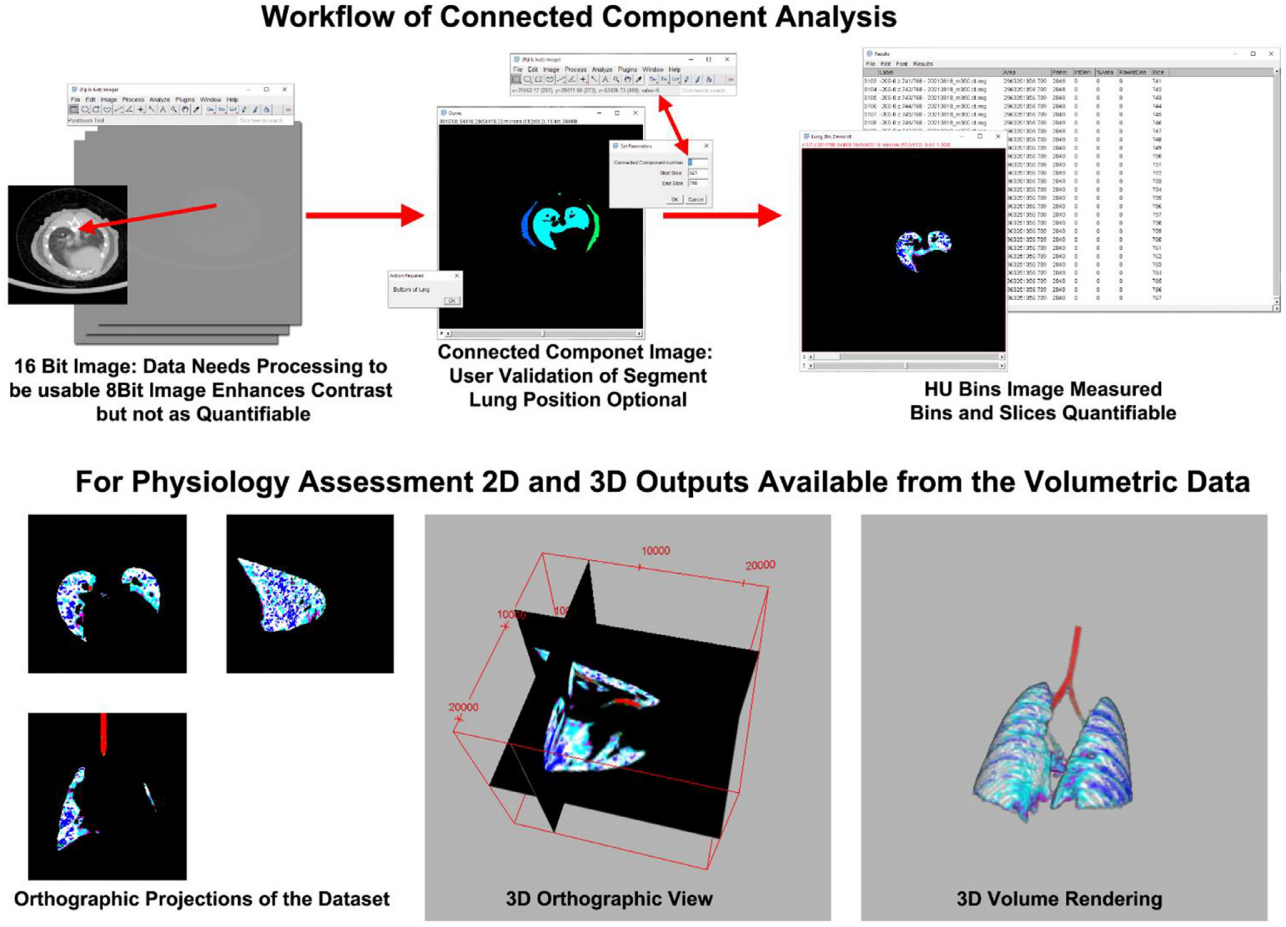
Top row, the workflow for converting 16bit data into a connected-component image (CC), and eventually quantified data. The CC image is used for the mask on the original data, and the user intervention step illustrated (Top Center) is the identification of the bottom (base) of the lung, and confirmation of the correct lung segmentation index, allowing progression of the analysis from the bottom (base) of the lung to the top (apex) or all the way up to the sinus cavity. The saved volumetric data can be reviewed in multiple formats including orthographic projection slice views (Bottom Left), 3D orthographic views (Bottom Center), and 3D volume rendering (Bottom Right).

In the image analysis, a rough threshold range of [−32,768, −200] HU was used to approximate the lung tissue density and create a segmentation mask. Note that the range [−200, 0] is connective tissue, but typically can result in non-specific segmentation. Fig. 3 shows that a connected-component analysis can be utilized to create labeled images in the script “ConnectedComponentsLung.ijm”. Manual Validation of lung area starts with running the script “CCMeasure.ijm" to run the macro "Measure_HU_From_CC_Image". In the user interaction/validation step, the lung region of interest is selected when the labeled component that matches the lung is verified, and the bottom (base) of the lung is identified. The binary mask of the labeled lung image is eroded to smooth the edges, fill holes in the binary mask, and include plural connective tissue around the lung. The original 16-bit float image is then segmented, by bin, with the lung mask, and the area is measured. The resulting measurements are exported as a .CSV file for further analysis at the user's discretion. When the bottom of the lung is located in the “Z” stack and the labeled image ID is verified, the code will continue to measure the original image with appropriate HU bins. In the labeled image, pointing at the correct label will display the index in the ImageJ status bar.

### Performance analysis and validation

**Fig. 4:**
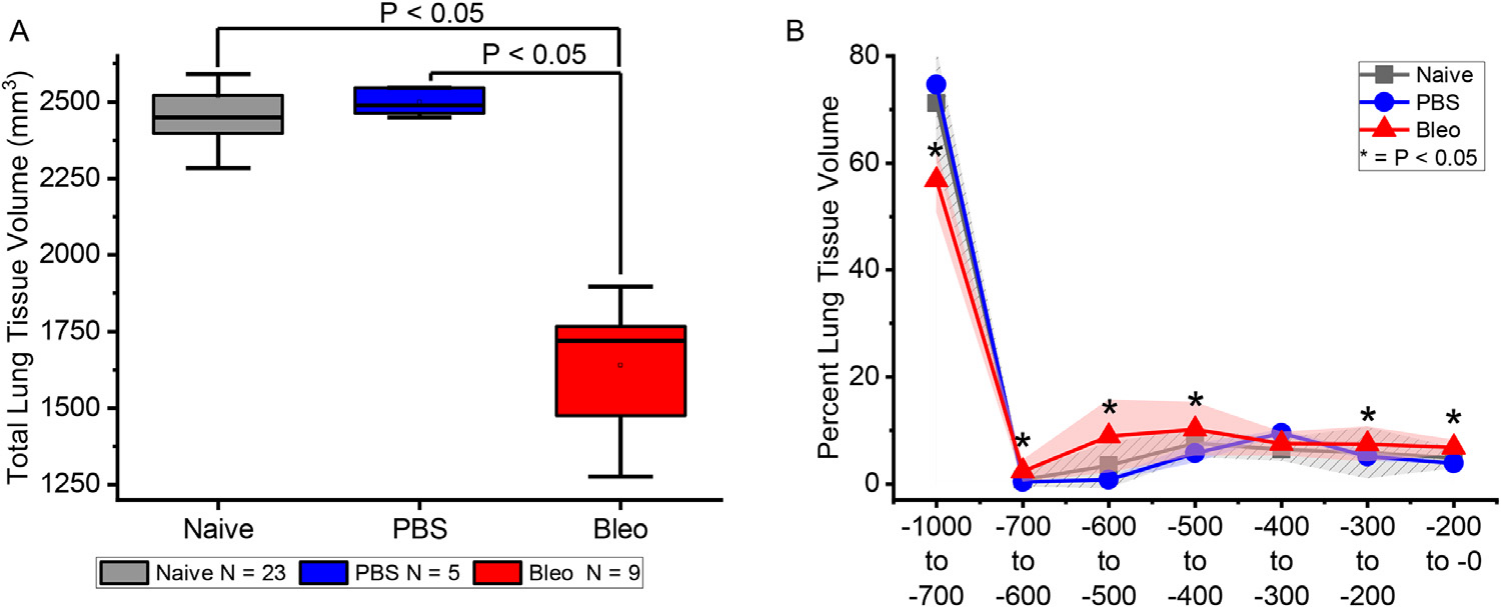
(A) The total lung tissue volume compared in naïve, PBS, and bleomycin (Bleo) exposed animals shows that bleomycin significantly decreased the total lung tissue volume as measured by microCT imaging, both as an overall volume measured variable, and (B) across HU tissue density bins expressed as a percent of total lung tissue volume. Data of box-whisker plot in panel A are represented as 75% confidence intervals with the horizontal line marking the median value, and outlier range indicated by the whiskers; Significant difference with bleomycin treated compared to both Naïve and PBS groups respectively, * *p* < 0.05 by ANOVA with Tukey's post-hoc test. Data of lung tissue volume across increasing density bins in Panel B are represented as means with group color-matching shaded 95% confidence intervals.

In the performance evaluation, naïve mice, phosphate buffered saline (PBS; vehicle control)-treated mice, and bleomycin-treated mice were assessed by microCT, with PBS and bleomycin exposures administered in small volumes (20 µL) intratracheally, under anesthesia. As shown in Fig. 4, the decreased lung volume and right shift of the histogram [22] were reproduced with the semi-automated connected-components method. Within the pooled untreated and pre-treatment groups of mice (labeled as naïve), the total lung tissue volume measurement averaged 2520±321 mm^3^ (Naïve; *n* = 23) and 2498±45 (PBS; *n* = 5) with no statistical difference between the groups (Fig. 4). Bleomycin treatment (Bleo; *n* = 9) significantly decreased the lung tissue size distribution by an average of 1637±212 mm^3^ of tissue volume, being statistically different as compared to both naïve and PBS groups (*P*<0.05 vs. Naïve and PBS). The semi-automated analysis reproduced the original findings; however, the efficiency was increased, as a decrease in user time, for the semi-manual segmentation, which was at a rate of 5–20 min per slice, as compared with the total user time for the semiautomatic full lung volume calculation (>500 slices), which was only 5 min. Thus, for reference, at a maximal rate of 5 min per slice for the semi-manual method, and typically 500 slices per lung, manual segmentation could take up to a week to process, given a assumption of uninterrupted 40 h work week. In computational time, on average, each connected-component volume was processed in less than half an hour, and analysis was completed in less than 5 min of user interaction for the entire volume of the lung. Digital reading and writing the 1GB+ microCT volumes were one of the major rate-limiting factors of the data analysis, but was completely computer speed dependent (hard drive read/write and processor time), proceeding automatically, thus and requiring no further user interaction.

**Fig. 5:**
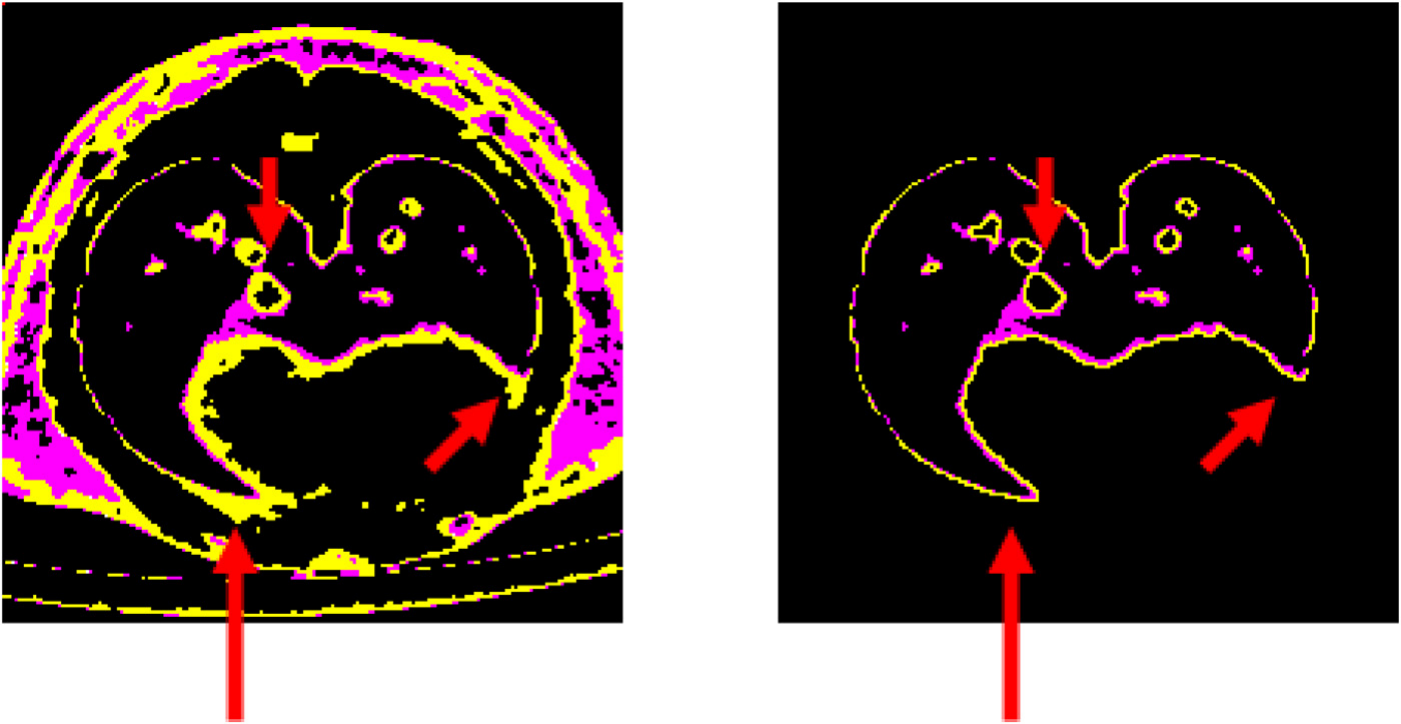
For comparison, the [−300 to −200, −200 to 0] HU bins (magenta and yellow channels respectively) are displayed from the reference image of the manual segmentation process (Left) and the output of the Semi-Automated method (Right). Red arrows landmark samples of key locations for the −200 to 0 HU (yellow) areas that can be overestimated by the reader when using the manual segmentation method. For example, as shown by the lower two arrows in the manual segmentation (left), cardiac connective tissue may be over-sampled and inadvertently read as lung tissue, in the segmented image, which is avoided using the semi-automated method which utilizes a sharper definition line for identification of the lung tissue boundary (right).

In the images shown in Fig. 5, the [−200 to 0] HU bins were the most variable in segmentation. In the case of manual segmentation, inclusion of the connective tissue around the pericardial cavity and lack of contrast with the pleural sac caused a discrepancy in the inclusion criteria, which was avoided with the semi-automated method. The manual method applied to all slices serially, and validation with histology sections could determine the discrepancy in airway measurements. It is worth noting that possible additional sources of propagated experimental error could come from oblique section of the CT images, or movement during scanning. In the studies presented here, the Siemens Inveon system that was used acquired the volume in 15 min without gating (synchronization of the image capture to the same point in the breathing cycle, i.e., max inhalation or expiration), whereas newer systems include gating, faster image acquisition, and higher spatial resolution. With newer systems, motion artifacts are reduced through increases in both the spatial and temporal resolutions, to provide more accurate data and higher resolution of the airway lumen.

### Agreement of manual and automated segmentation

Six individual lung image analysts were provided a step-by-step instruction sheet to guide their work through the steps of the manual image identification and segmentation process. According to the instructions, the analysts segmented 10 selected sample slices from the lung images, and the average measurements were utilized to determine the agreement. Each manual segmentation (training set) was interpolated (applying the ROI to two additional images offset by ± 5 slices) as a data augmentation step to compare regional variance and agreement. In the test design, an expert analysist (Analyst1, trained on segmenting multiple CT datasets) was matched with 5 additional analysts, who were novice to experienced users that tested the system for reproducibility.

**Fig. 6:**
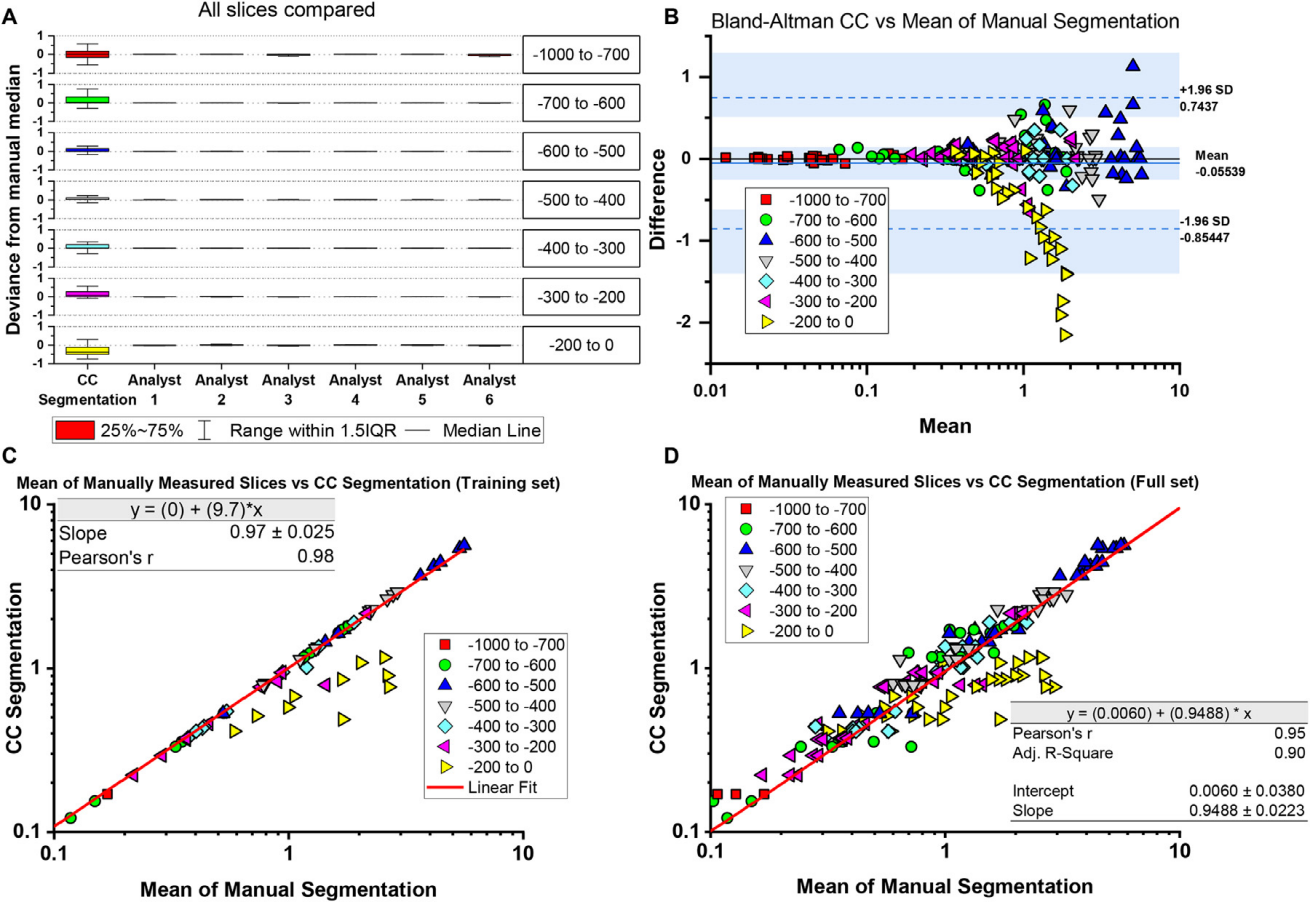
Plots of the segmentation area (voxels in mm^3^) are compared. (A) The deviance from manual segmentation was measured and plotted as box plots for the Connected-Components (CC) method as compared with the readings from each Analyst 1–6; as shown, overall variance was low within the dataset. (B) Using a Bland-Altman plot, the CC segmentation and manual segmentation was compared, showing an overall high agreement within the confidence interval (boundaries shown by dashed blue lines). (C) The test set was compared to CC segmentation with linear regression obtaining a slope of 0.97, which is very close to 1.0, and a Pearson's R of 0.98, which indicate a strong linear relationship and agreement between the methods. (D) When expanded to show the full set of data, the slope of 0.95 and Pearson's R of 0.95 was obtained, therefore maintaining both the strong agreement and linear relationship between the two methods.

The manual segmentation assessment set and the full set of slices (manual and interpolated) was compared to the matching slices in the semi-automated connected-components segmentation, as shown in Fig. 6c. In the fitting analysis, the CC segmentation, as compared to the mean of 6 manual segmentations, forms a strong linear relationship with the Pearson's R of 0.98, a slope of 0.97 (*p* < 0.05; ANOVA, indicating that the slope was significantly different from 0), and an intercept approaching the origin of the plot (0,0). In the subsequent analysis of the full set of data, manual and interpolated slices were compared to CC segmented slices, and a near-linear fit was obtained with the slope of 0.95 (*P* < 0.05; ANOVA), Pearson's R of 0.95, and a y-intercept of 0.006, again nearly at the origin of the plot (0,0). The Bland-Altman plot shows that the [−200 to 0] HU bin range is the only notable departure from the confidence interval; however, this was expected with the potential for overestimation with the connective tissue around the pericardium, as suggested above, and illustrated in Fig. 5. Thus, the noticeable divergence in the linear fitting is in the measurement of the [−200 to 0] HU bin-associated data (yellow triangles), where, as expected, variance in inclusion and potential overestimation of the manual segmentation leads to a lack of agreement not observed in the other density bins measured.

Our software macro was designed to be simple and stable, from within a widely available free source. While commercially available systems may have better computational optimizations and specific purpose-built interfaces, this general approach is open-sourced to be adaptable for a variety of users. Proprietary systems, such as Amira or MATLAB, provide powerful analytical tools and professional rendering, but require subscriptions. All algorithms and tools used in our segmentation are established in open-source or public domain code. While we acknowledge that additional refinements of lung tissue density quantification around the cardiac space can be made, the methodology produces deterministic results that may be adapted or improved for specific cases.

In conclusion, we developed a semi-automated method of lung imaging analysis that can (1) reliably quantify differences between controls and experimental lung injury models, (2) deliver deterministic results using an unbiased and parameter free process with reduced user variance, (3) reduce user time-on-task and increase throughput in data analysis, and (4) offer a portable and cross-platform approach based on the ImageJ macro. With ever increasing throughput for data collection and the need for un-biased analysis, this methodology aims to reduce user interaction and still provide rigorous analysis. By sharing the code, we aim to help increase the efficiency and reproducibility of image analyses, for investigators who may be interested in utilizing a freely-available semi-automated methodology for their image data analyses.

## Funding

This work was supported by the NIEHS T32 Training Grant T32ES007254, and the Brown Foundation.

## Ethics statements

All experiments were done with approval of the University of Texas Medical Branch (UTMB) Institutional Animal Care and Use Committee (IACUC) and adhered to guidelines set by National Institutes of Health (NIH) and Association for Assessment and Accreditation of Laboratory Animal Care (AAALAC). Male and Female C57BL/6 J mice (The Jackson Laboratory https://www.jax.org/) were used in these studies.

## CRediT authorship contribution statement

**Jonathan D. Luisi:** Conceptualization, Methodology, Formal analysis, Writing – original draft, Project administration, Software, Validation, Data curation, Visualization. **Jonathan L. Lin:** Validation, Investigation, Writing – review & editing. **Lorenzo F. Ochoa:** Validation, Investigation, Writing – review & editing. **Ryan J. McAuley:** Validation, Investigation, Writing – review & editing. **Madison G. Tanner:** Validation, Investigation, Writing – review & editing. **Obada Alfarawati:** Validation, Investigation, Writing – review & editing. **Casey W. Wright:** Writing – review & editing, Resources. **Gracie Vargas:** Writing – review & editing, Resources. **Massoud Motamedi:** Writing – review & editing, Resources. **Bill T. Ameredes:** Conceptualization, Methodology, Formal analysis, Writing – original draft, Project administration, Visualization, Supervision, Resources, Funding acquisition.

## Declaration of Competing Interest

The authors declare that they have no known competing financial interests or personal relationships that could have appeared to influence the work reported in this paper.

## Data Availability

Code made available on Github

## Appendix Supplementary materials

Image, application 1
