# Supplementary material for: Semi-automated micro-computed tomography lung segmentation and analysis in mouse models
Source: MethodsX. 2023 Apr 20;10:102198. doi: 10.1016/j.mex.2023.102198 (PMC10154963; doi:10.1016/j.mex.2023.102198)
Supplement: Supplementary file 1 [file mmc1.docx]

**Supplementary material *and/or* additional information [OPTIONAL]**

Code available from GitHub repository: <https://github.com/UTMB-Luisi/SemiAutomated_Lung_CT>

File “CT_AutoHU_SelectBin.ijm” to select the radiodensity bins by preset HU ranges or a custom range:

**macro** **"** **CT_AutoHU_SelectBin.ijm "** {

Dialog.create**("HU Bin Modes");**

Method **=** newArray**("Default",** **"MOD",** **"Range","Cancel");**

Dialog.addRadioButtonGroup**("Select HU Bin",** Method**,** 4**,** 1**,** **"Default");**

Dialog.addNumber**("Min",** -1000**);**

Dialog.addNumber**("Max",** 1**);**

Dialog.addNumber**("Bin",** 200**);**

Dialog.show**();**

method **=** Dialog.getRadioButton**();**

**if** **(**method **==** **"Cancel")** {

exit**("Canceled");**

}

min **=** Dialog.getNumber**();**

max **=** Dialog.getNumber**();**

bin **=** Dialog.getNumber**();**

list **=** getList**("image.titles");**

**if** **(**list.length**==**0**)**{

// get dir

tmpdir**=** getDirectory**("Choose a Directory");**

**if** **(!**File.exists**(**tmpdir**))**

{

exit**();**

// directory error no point

}

list **=** getFileList**(**tmpdir**);**

setBatchMode**(true);**

OutputDir **=** tmpdir**+"Output_Vol_tiff"+**File.separator**;**

**if** **(!**File.exists**(**OutputDir**))**{

File.makeDirectory**(**OutputDir**);**

//print("Directory Created", OutputDir);

}

counter **=** 0**;**

print **("File List size= "** **+** list.length**);**

**for** **(**i**=**0**;** i**<**list.length**;** i**++)**{

//convert .oct files

**if** **(**endsWith**(**list**[**i**],** **".hdr"))**{

print**("file "** **+** list**[**i**]** **+** **" number: "** **+** i**);**

run**("Bio-Formats Importer",** **"open=["** **+** tmpdir **+** list**[**i**]** **+** **"] color_mode=Default rois_import=[ROI manager] view=Hyperstack stack_order=XYCZT");**

**if** **(**method **==** **"Default")** {

setDefaultBin**();**

}

**else** **if** **(**method **==** **"MOD")** {

setModBin**();**

} //if mod

**else**{

HUbinRange**(**min**,** max**,** bin**);**

}

name **=** replace**(**list**[**i**],** **".ct.img.hdr",** **".tiff");**

saveAs**("Tiff",** OutputDir **+** method **+"_"** **+** name**);**

close**();**

}

}

}**else** {

**if** **(**method **==** **"Default")** {

setDefaultBin**();**

} //if default

**else** **if** **(**method **==** **"MOD")** {

setModBin**();**

} //if mod

**else**{

HUbinRange**(**min**,** max**,** bin**);**

} //set by range

} //end signle image

print**("finished: ");**

} //end macro

**function** setDefaultBin**()**{

myImageID **=** getImageID**();**

imgName **=** getTitle**();**

HU_Bin**(**myImageID**,** 1**,** -1000**,** -500**);**

HU_Bin**(**myImageID**,** 2**,** -500**,** -400**);**

HU_Bin**(**myImageID**,** 3**,** -400**,** -300**);**

HU_Bin**(**myImageID**,** 5**,** -300**,** -200**);**

HU_Bin**(**myImageID**,** 6**,** -200**,** 0**);**

selectImage**(**myImageID**);**

run**("Enhance Contrast...",** **"saturated=0 process_all use");**

run**("8-bit");**

rename**("C4");**

run**("Channels Tool...");**

run**("Merge Channels...",** **"c1=C1-HU-1000to-500 c2=C2-HU-500to-400 c3=C3-HU-400to-300 c4=C4 c5=C5-HU-300to-200 c6=C6-HU-200to0 create");**

rename**(**imgName**);**

}

**function** HUbinRange**(**min**,** max**,** bin**)** {

myImageID **=** getImageID**();**

imgName **=** getTitle**();**

print**("\\Clear");**

HUStart **=** min**;**

channel **=** 1**;**

channelNameList **=** **"";**

**while** **(**HUStart **+** bin **<** max**)** {

//

**if** **(**channel **!=** 4**)** {

HUEnd **=** HUStart **+** bin**;**

HU_Bin**(**myImageID**,** channel**,** HUStart**,** HUEnd**);**

ChannelName **=** **"C"+** channel **+** **"-HU"** **+** HUStart **+** **"to"** **+** HUEnd**;**

channelNameList **=** channelNameList **+** **"c"** **+** channel **+** **"="** **+** ChannelName **+** **" ";**

print**(**channelNameList**);**

HUStart **=** HUEnd**;**

}**else** {

// C4

channelNameList **=** channelNameList **+** **"c4=C4 ";**

}

channel **=** channel +1**;**

} //end while

selectImage**(**myImageID**);**

run**("Enhance Contrast...",** **"saturated=0 process_all use");**

run**("8-bit");**

rename**("C4");**

run**("Channels Tool...");**

run**("Merge Channels...",** channelNameList **+** **" create");**

rename**(**imgName**);**

} //end function hubinrange

**function** setModBin**()**{

myImageID **=** getImageID**();**

imgName **=** getTitle**();**

HU_Bin**(**myImageID**,** 1**,** -1000**,** -700**);**

HU_Bin**(**myImageID**,** 2**,** -700**,** -600**);**

HU_Bin**(**myImageID**,** 3**,** -600**,** -500**);**

HU_Bin**(**myImageID**,** 4**,** -500**,** -400**);**

HU_Bin**(**myImageID**,** 5**,** -400**,** -300**);**

HU_Bin**(**myImageID**,** 6**,** -300**,** -200**);**

HU_Bin**(**myImageID**,** 7**,** -200**,** 0**);**

run**("Channels Tool...");**

run**("Merge Channels...",** **"c1=C1-HU-1000to-700 c2=C2-HU-700to-600 c3=C3-HU-600to-500 c4=C4-HU-500to-400 c5=C5-HU-400to-300 c6=C6-HU-300to-200 c7=C7-HU-200to0 create");**

rename**("mod_"** **+**imgName**);**

}

**function** HU_Bin**(**myImageID**,** channel**,** HUStart**,** HUEnd**)** {

selectImage**(**myImageID**);**

run**("Duplicate...",** **"duplicate");**

setThreshold**(**HUStart**,** HUEnd**);**

run**("Convert to Mask",** **"method=Default background=Light black");**

rename**("C"+** channel **+** **"-HU"** **+** HUStart **+** **"to"** **+** HUEnd**);**

}

File “Measure HU.ijm”

**macro** **"MeasureHU"** {

run**("Clear Results");**

imageName **=** getTitle**();**

imageID **=** getImageID**();**

dir **=** getDirectory**("image");**

Stack.getDimensions**(**iWidth**,** iHeight**,** iChannels**,** iMAXslice**,** iFrames**);**

**if** **(**iWidth **!=** 200**)** {

//crop

makeRectangle**(**207**,** 127**,** 200**,** 200**);**

setTool**("rectangle");**

waitForUser**("Select Crop Area");**

roiManager**("Add");**

roinum **=** roiManager**("count");**

roiManager**("Select",** roinum -1**);**

roiManager**("Rename",** **"Crop_"** **+** imageName**);**

run**("Crop");**

}

Stack.getPosition**(**channel**,** slice**,** frame**);**

OrthoProjecter**(**imageID**,** slice**,** dir**);**

selectImage**(**imageID**);**

Stack.setSlice**(**slice**);**

run**("Stack to RGB",** **"keep");**

//run("Duplicate...", "duplicate slices=" slice);

//run("Stack to RGB");

setTool**("wand");**

run**("Wand Tool...",** **"tolerance=3 mode=4-connected");**

//run("Properties... ", " stroke=red width=.5");

waitForUser**("select area to measure");**

roiManager**("Add");**

roinum **=** roiManager**("count");**

roiManager**("Select",** roinum -1**);**

roiName **=** **"slice"** **+** slice **+** **"_"** **+** imageName**;**

roiManager**("rename",** roiName**);**

roiManager**("save selected",** dir **+** roiName **+** **".roi");**

saveAs**("TIFF",** dir **+** roiName**);**

selectWindow**(**imageName**);**

run**("Set Measurements...",** **"area perimeter integrated area_fraction stack display redirect=None decimal=3");**

MeasureSlice**(**imageID**,** slice**);**

print**(**imageName **+** **"\t slice: \t"** **+** slice**);**

MeasureSlice**(**imageID**,** slice -5**);**

MeasureSlice**(**imageID**,** slice **+** 5**);**

saveAs**("Results",** dir **+** **"slice_"** **+** slice **+** **"_"** **+** imageName **+** **".csv");**

}

**function** OrthoProjecter**(**imageID**,** sliceID**,** directory**)** {

selectImage**(**imageID**);**

imgName **=** getTitle**();**

Stack.getDimensions**(**width**,** height**,** channels**,** MAXslice**,** frames**);**

run**("Orthogonal Views");**

//selectImage(imageID);

Stack.setOrthoViews**(**floor**(**width/2**),** floor**(**height/2**)** **,** sliceID**);**

selectImage**(**imageID**);**

list **=** getList**("image.titles");**

// print( list.length);

**for** **(**i**=**0**;** i**<**list.length**;** i**++)**{

//print("testing: " + list[i]);

**if** **(** startsWith**(**list**[**i**]** **,** **"YZ"))**

{

YZ **=** list**[**i**];**

//print ("found " + YZ);

}

**else** **if** **(** startsWith**(**list**[**i**]** **,** **"XZ"))**

{

XZ **=** list**[**i**];**

//print ("found " + XZ);

}

}

selectWindow**(**YZ**);**

run**("Overlay Options...",** **"stroke=yellow width=3 fill=none set apply");**

run**("Flatten");**

YZ **=** getTitle**();**

selectWindow**(**XZ**);**

run**("Overlay Options...",** **"stroke=yellow width=3 fill=none set apply");**

run**("Flatten");**

XZ **=** getTitle**();**

selectWindow**(**imgName**);**

run**("Overlay Options...",** **"stroke=yellow width=3 fill=none set apply");**

run**("Flatten",** **"slice");**

flat **=** getTitle**();**

run**("Combine...",** **"stack1=["** **+** flat **+** **"] stack2=["** **+** YZ **+** **"]");**

run**("Combine...",** **"stack1=[Combined Stacks] stack2=["** **+** XZ **+** **"] combine");**

run**("Subtract...",** **"value=20");**

saveAs**("TIFF",** directory **+"Ortho_slice-"** **+** sliceID **+** **"-"** **+** imgName**);**

}

**function** MeasureSlice**(**imageID**,** slice**)** {

selectImage**(**imageID**);**

imageName **=** getTitle**();**

//Stack.setSlice(slice);

run**("Duplicate...",** **"title="** **+** slice **+** **"_"** **+** imageName **+** **" duplicate slices="** **+** slice**);**

sliceID **=** getImageID**();**

roinum **=** roiManager**("count");**

roiManager**("Select",** roinum -1**);**

setBackgroundColor**(**0**,** 0**,** 0**);**

run**("Clear Outside");**

run**("Select None");**

Stack.getDimensions**(**iWidth**,** iHeight**,** iChannels**,** iMAXslice**,** iFrames**);**

**for** **(**i **=** 1**;** i **<=** iChannels**;** i**++)** {

selectImage**(**sliceID**);**

Stack.setChannel**(**i**);**

run**("Measure");**

}

}

File “ConnectedComponentsLung.ijm”

**macro** **"ConnectedLungRegions"** {

list **=** getList**("image.titles");**

**if** **(**list.length**==**0**)**{

// get dir

tmpdir**=** getDirectory**("Choose a Directory");**

**if** **(!**File.exists**(**tmpdir**))**

{

exit**();**

// directory error no point

}

list **=** getFileList**(**tmpdir**);**

setBatchMode**(true);**

OutputDir **=** tmpdir**;**

counter **=** 0**;**

print **("File List size= "** **+** list.length**);**

**for** **(**i**=**0**;** i**<**list.length**;** i**++)**{

//convert

**if** **(**endsWith**(**list**[**i**],** **".hdr"))**{

getDateAndTime**(**year**,** month**,** week**,** day**,** hour**,** min**,** sec**,** msec**);**

print**("file "** **+** list**[**i**]** **+** **" number: "** **+** i **+** **"Time"+**toString**(**hour**)+":"+**toString**(**min**));**

startTime **=** getTime**();**

run**("Bio-Formats Importer",** **"open=["** **+** tmpdir **+** list**[**i**]** **+** **"] color_mode=Default rois_import=[ROI manager] view=Hyperstack stack_order=XYCZT");**

CCImage**();**

name **=** replace**(**list**[**i**],** **".ct.img.hdr",** **".tiff");**

saveAs**("Tiff",** OutputDir **+** **"CC_"** **+** name**);**

close**();**

stopTime **=** getTime**();**

print**("CC RunTime: "** **+** **(**stopTime **-** startTime**)**/1000**);**

}

}

}**else** {

imgName **=** getTitle**();**

CCImage**();**

rename**(**imgName **+** **"_CC");**

} //end signle image

//print("finished: " + getTime()/1000);

getDateAndTime**(**year**,** month**,** week**,** day**,** hour**,** min**,** sec**,** msec**);**

print**("Finished at Time"+**toString**(**hour**)+":"+**toString**(**min**));**

} //end macro

**function** CCImage**()** {

//imgName = getTitle();

run**("Duplicate...",** **"duplicate");**

setThreshold**(**-32768**,** -200**);**

run**("Convert to Mask",** **"method=Default background=Light black");**

myImageID **=** getImageID**();**

Stack.getDimensions**(**width**,** height**,** channels**,** MAXslice**,** frames**);**

print**("frames "** **+** frames**);**

slice **=** 1**;**

setSlice**(**slice**);**

**do** {

selectImage**(**myImageID**);**

doWand**(**5**,** 5**);**

floodFill**(**5**,** 5**);**

slice **=** slice **+** 1**;**

setSlice**(**slice**);**

}**while** **(**slice **<** MAXslice **);**

//print("max: " + MAXslice + " Slice" + slice);

run**("Find Connected Regions",** **"allow_diagonal display_one_image display_results regions_for_values_over=100 minimum_number_of_points=10000 stop_after=-1");**

//rename(imgName + "_CC");

}

File “CCMeasure.ijm”

**macro** **"Measure_HU_From_CC_Image"** {

setSlice**(**500**);**

Stack.getDimensions**(**iWidth**,** iHeight**,** iChannels**,** iMAXslice**,** iFrames**);**

waitForUser**("Bottom of lung");**

Stack.getPosition**(**channel**,** slice**,** frame**);**

getStatistics**(**area**,** mean**,** min**,** max**,** std**,** histogram**);**

//print(min +" " + max);

Dialog.create**("Set Parameters");**

Dialog.addNumber**("Connected Component number",** max**);**

Dialog.addNumber**("Start Slice ",** slice**);**

Dialog.addNumber**("End Slice",** iMAXslice**);**

Dialog.show**();**

CC **=** Dialog.getNumber**();**

startSlice **=** Dialog.getNumber**();**

endSlice **=** Dialog.getNumber**();**

//CC = getNumber("CC to keep: ", max);

setThreshold**(**CC**,** CC**);**

run**("Convert to Mask",** **"method=Default background=Light black");**

run**("Erode",** **"stack");**

//run("Fill Holes", "stack");

CC_id **=** getImageID**();**

CC_Name **=** getTitle**();**

path **=** getDirectory**("Image");**

CT_Image **=** replace**(**CC_Name**,** **"CC_",** **"");**

CT_Image **=** path **+** replace**(**CT_Image**,** **".tiff",** **".ct.img.hdr");**

print**(**CT_Image**);**

**if** **(**File.exists**(**CT_Image**)** **)**{

run**("Bio-Formats Importer",** **"open=["** **+** CT_Image **+** **"] color_mode=Default rois_import=[ROI manager] view=Hyperstack stack_order=XYCZT");**

}**else** {

rawFile **=** File.openDialog**("Select CT Data");**

run**("Bio-Formats Importer",** **"open=["** **+** rawFile **+** **"] color_mode=Default rois_import=[ROI manager] view=Hyperstack stack_order=XYCZT");**

}

CT_ID **=** getImageID**();**

HU_Bin**(**CT_ID**,** CC_Name**,** -1000**,** -700**,** startSlice**,** endSlice**);**

HU_Bin**(**CT_ID**,** CC_Name**,** -700**,** -600**,** startSlice**,** endSlice**);**

HU_Bin**(**CT_ID**,** CC_Name**,** -600**,** -500**,** startSlice**,** endSlice**);**

HU_Bin**(**CT_ID**,** CC_Name**,** -500**,** -400**,** startSlice**,** endSlice**);**

HU_Bin**(**CT_ID**,** CC_Name**,** -400**,** -300**,** startSlice**,** endSlice**);**

HU_Bin**(**CT_ID**,** CC_Name**,** -300**,** -200**,** startSlice**,** endSlice**);**

HU_Bin**(**CT_ID**,** CC_Name**,** -200**,** 0**,** startSlice**,** endSlice**);**

name **=** replace**(**CC_Name**,** **".tiff",** **".csv");**

saveAs**("Results",** path **+** name**);**

run**("Merge Channels...",** **"c1=-1000--700 c2=-700--600 c3=-600--500 c4=-500--400 c5=-400--300 c6=-300--200 c7=-200-0 create");**

//run("Channels Tool...");

run**("Make Composite");**

saveAs**("Tiff",** path **+** **"Lung_Bin_"** **+** CC_Name**);**

}

**function** HU_Bin**(**CT_ID**,** CC_Name**,** HUStart**,** HUEnd**,** sliceStart**,** sliceEnd**)** {

selectImage**(**CT_ID**);**

run**("Duplicate...",** **"duplicate");**

//setThreshold(-32768, -700);

setThreshold**(**HUStart**,** HUEnd**);**

run**("Convert to Mask",** **"method=Default background=Light black");**

Bin_ID **=** getImageID**();**

Bin_Name **=** getTitle**();**

imageCalculator**("AND create stack",** Bin_Name**,** CC_Name**);**

Mask_ID **=** getImageID**();**

rename**(**HUStart **+** **"-"** **+** HUEnd**);**

//run("Set Measurements...", "area perimeter area_fraction stack display redirect=None decimal=3");

run**("Set Measurements...",** **"area perimeter integrated area_fraction stack display redirect=None decimal=3");**

**for(**i **=** sliceStart**;** i **<** sliceEnd**;** i**++)**{

setSlice**(**i**);**

run**("Measure");**

}

selectImage**(**Bin_ID**);**

close**();**

}
